# Eat Healthy, Be Active Community Workshops implemented with rural Hispanic women

**DOI:** 10.1186/s12905-020-01157-5

**Published:** 2021-01-13

**Authors:** Janeth I. Sanchez, Katherine J. Briant, Samantha Wu-Georges, Virginia Gonzalez, Avigail Galvan, Sara Cole, Beti Thompson

**Affiliations:** 1 Department of Health Services, University of Washington School of Public Health, Box 357230, Seattle, WA 98195 USA; 2 Fred Hutchinson Cancer Research Center, 1100 Fairview Ave. N., M3-B232, Seattle, WA 98166 USA; 3 Fred Hutchinson Cancer Research Center - Center for Community Health Promotion, 320 N. 16th Street, Sunnyside, WA 98944 USA

**Keywords:** Obesity, Nutrition, Physical activity, Hispanics, *Promotoras*

## Abstract

**Background:**

In the U.S., obesity disproportionately affects some racial/ethnic groups more than others; 42.5% of Hispanic adults are obese, compared to 32.6% of non-Hispanic whites (NHW). Research also shows that Mexican American women are 40% more likely to be overweight, as compared to NHW women. With high obesity rates among Hispanics, improving healthier lifestyle practices is an important step for reducing health disparities. The Eat Healthy, Be Active (EHBA) community workshops were developed to assist individuals in translating national nutrition and physical activity recommendations into action. *Promotora-led* EHBA workshops could be used to promote obesity-related health behavior lifestyle changes among Hispanics.

**Methods:**

Hispanic women from rural communities in Washington state were recruited to participate in a six-week *Promotora*-led workshop series. This pilot study used a pre- and post-test study design to examine differences in healthy lifestyle knowledge and practices.

**Results:**

A total of 49 Hispanic women participated in the workshops, of whom 45% were obese. Six-weeks after implementation of EHBA, women had improvements in healthy lifestyle practices, including an increase in nutrition label literacy, decrease in consumption of food eaten in restaurants, and an increase in the number of times a woman performed physical activity long enough to make them sweat.

**Conclusion:**

The findings from this pilot study indicate that delivering EHBA workshops through *promotoras* is a feasible culturally relevant approach to promoting healthier lifestyle practices among Hispanic women. Further, focusing on females, who do the food shopping and preparation in their homes, may help increase awareness among whole families.

## Background

In the US, over 15 million American adults are obese [1]. Data from the National Health and Nutrition Evaluation Survey (NHANES), indicate that obesity has been steadily increasing over the past few decades and by 2030, obesity rates are expected to rise by 33% [2, 3]. Especially alarming is the increasing significant positive linear trend in obesity rates among Hispanic women who demonstrate a 10% higher prevalence of obesity compared to non-Hispanic white (NHW) women [4–6]. Obesity is a major public health concern for Hispanic women as it is a significant risk factor for the development of serious chronic conditions, such as coronary heart disease (CHD), diabetes, and cancer [7–9]. For example, although Hispanics have lower prevalence of CHD compared to NHWs, Hispanic women are likely to develop heart disease ten years earlier than NHW women and are less likely to be aware of their risk for CHD [10, 11]. Similarly, studies have consistently demonstrated an increasing prevalence of diabetes and cancer associated with increasing weight among Hispanic women [12–14]. Obesity increases the risk for developing breast, colorectal, and endometrium cancer among Hispanic women and has been linked to poorer breast cancer survival in this population [15, 16]. Maintaining a healthy weight and engaging in regular physical activity are important approaches that can help reduce incidence and mortality from CHD, diabetes, and cancer [8, 15].

Educating Hispanic women about obesity-related risk factors and the importance of adopting healthy behaviors is important since even a loss of 3% to 5% in weight can result in measurable improvement in managing chronic conditions and comorbidities [17, 18]. Increasing knowledge and awareness of risk factors and promotion of healthier lifestyles are key components of behavioral health interventions. Further, obesity-related health promotion efforts focusing on increasing consumption of healthier food options and increasing physical activity are of importance among sub-populations of Hispanic women who experience higher prevalence of diabetes, such as Mexican–American women [6].

Unfortunately, Hispanic women, especially those residing in rural geographical areas, continue to face significant barriers that may limit the ability to achieve desired health outcomes. For example, rural Hispanic communities face several healthcare challenges, including shortages in healthcare-related services and personnel, as well as a lack of access to culturally appropriate resources and information, that aggravate obesity incidence and contribute to poor obesity-related outcomes [19]. The lack of community resources in rural areas (e.g., poor infrastructure to encourage physical activity, lack of access to financially accessible healthy foods at local supermarkets) further exacerbate poor health outcomes among Hispanic women [19–21]. Identifying and implementing culturally tailored health promotion interventions may assist in improving obesity-related outcomes among Hispanic women living in rural areas.

To address such barriers, trends in the field of health promotion have shifted from clinically delivered individual interventions to efforts that use a community-based approach that encompasses social and environmental factors to deliver interventions in community settings [22–24]. Research suggests that community-based interventions promote healthy lifestyle behaviors among Hispanic women [23, 25]; for example, weight management programs delivered in community settings have proven to be effective in promoting weight loss, improving obesity-related risk behaviors, and decreasing BMI among Hispanic women immigrants [26].

Moreover, *promotora*-delivered interventions promote healthier dietary habits and increased performance of physical activity among Hispanic women [26–28]. The WISEWOMAN program, for example, utilized a *promotora*-led approach to recruit, educate and deliver a lifestyle health promotion program among Hispanic women living in California. Women receiving the intervention demonstrated improved eating habits and physical activity, as well as a reduced 10-year CHD risk [29]. Another example, the ENLACE program, was a multisite research study aimed to deliver a community-based physical activity intervention to Hispanic women using a *promotora*-led delivery model [30]. Hispanic women who received the ENLACE intervention increased their physical activity by 56.4% compared to 48.8% among the women in the control group [30].

Much of the success of *promotora*-led behavioral interventions among Hispanic women is linked to the *promotoras’* ability to be culturally sensitive and to lead and motivate participants with culturally relevant tools, knowledge, and support [28]. *Promotoras* appear to serve as cultural mediators and role models who provide emotional and social support in ways that make Hispanic women feel secure and comfortable, thus facilitating acquisition of the skills that enable healthy lifestyle changes [28, 31]. Overall, findings from systematic reviews of *promotora*-led interventions provide evidence of their effectiveness in improving health-related behaviors and outcomes [32–34].

Given the burden of obesity-related outcomes faced by Hispanic women residing in rural areas, there is a continued need for community-based *promotora*-led health promotion interventions that are culturally adapted and tailored for rural populations. One such community-based program, the *Eat Healthy, Be Active Community Workshops* (EHBA) was developed by the U.S. Department of Health and Human Services’ Office of Disease Prevention and Health Promotion. The focus of EHBA is to educate and assist individuals from varying literacy levels and socioeconomic backgrounds in gaining skills to adopt and sustain recommended nutrition and physical activity behaviors to promote good health and reduce risk of chronic diseases [35]. The curriculum has been tested among diverse populations in the United States; however, no study to date has evaluated its effectiveness among rural Hispanics. The aim of the pilot study was to assess the feasibility of implementing a *promotora*-led EHBA intervention to improve obesity-related behaviors among Hispanic women residing in a predominantly Hispanic rural community.

## Methods

### Setting

The Lower Yakima Valley of Washington State (the Valley) is a region known for its vast agricultural industry, rurality, and predominant Hispanic population; approximately 68% of the population in the Valley is Hispanic and the majority are of Mexican descent [36]. Hispanics living in the Valley have lower socioeconomic status, lower educational attainment and limited access to health care services that contribute to poor health outcomes [37].

### Recruitment and participation

The target for this pilot intervention was 50 Hispanic women [38], 18 years or older, who were responsible for the food shopping and food preparation in their homes. To recruit individuals, flyers were placed in various locations throughout the Valley, including at grocery and convenience stores, and community centers. Individuals were also recruited at local community events, including health fairs in the region. Once recruited, participants were registered to participate in one of the workshop series held in 2015. Of the 50 women who agreed to participate, two women did not complete the follow-up questionnaire. In addition, the number of women who responded to each item varied from baseline to follow-up.

### Identifying an intervention program

Researchers in the Valley use community-based participatory research (CBPR) methods to address health disparities experienced by the Hispanic population. A Community Advisory Board (CAB) in the Valley noted the high prevalence of obesity in the region and recommended that a research effort be initiated to address obesity rates in the community. The CAB recommended that a *promotora* approach be part of the intervention. After a search for validated programs, we identified the *Eat Healthy, Be Active Community Workshops* (EHBA) program as being appropriate for the intervention [35].

### EHBA intervention

The *Eat Healthy, Be Active Community Workshops* (EHBA) is an educational curriculum developed in English and Spanish to promote consumption of healthy balanced diets and increased physical activity [35]. The curriculum was designed using low-literacy principles and pilot tested at ten different sites across the US, including state cooperative extensions, faith-based organizations, worksite wellness programs, and parent groups. EHBA workshops consist of six one-hour interactive workshops and include the following sections: 1) *Eating Healthy Food That Tastes Great*, 2) *Quick, Healthy Meals and Snacks*, 3) *Eating Healthy on a Budget*, 4) *Tips for Losing Weight and Keeping it Off*, 5) *Making Healthy Eating Part of Your total Lifestyle*, and 6) *Physical Activity is Key to Living Well*. The EHBA curriculum includes a lesson plan, learning objectives for each workshop, talking points, hand-on-activities, videos and handouts. The workshops may be taught by various trained professionals, including *promotores,* and may take place in a variety of community settings.

### Training

The Fred Hutchinson Cancer Research Center’s (Fred Hutch) Center for Community Health Promotion (CCHP) has a Community Health Educator (CHE) whose role is to train local community health workers (called *promotores*) to implement health promotion interventions among local Hispanic groups or individuals in the Valley. The cadre of *promotores* are trusted individuals from the Valley who are bilingual and bicultural. The *promotores* are trained in community-based participatory research (CBPR), collecting survey data, and facilitating educational interventions and community outreach. The CHE trained six *promotores* to implement the EHBA. Each promotor/a received a two-day training in which they went over all six workshops, engaged in role-playing to answer questions, discussed various features of the program, and obtained certification in the content of the curriculum. For this pilot, EHBA was facilitated and implemented by two female *protomoras* of those trained.

### Implementation

We offered the EHBA workshop series at five different times between January 2015 and December 2015. Participants in the workshops were consented and were asked to complete a pre-test (baseline) at the beginning of Workshop One. Each session began with an Icebreaker activity and an introduction explaining the purpose of the workshop and review of the learning objectives. *Promotores* reviewed specific workshop-related talking points, handouts, presented a video and facilitated activities (i.e., MyPlate drawing, how much sugar in a soda?, Using a slow cooker) during each session. Participants were asked to complete a post-test (follow-up) at the end of the six-week series.

### Study design and measures

For this project, a pre- and post-test study design was used to assess the feasibility of implementing EHBA among Hispanic women and its ability to change obesity-related behaviors from baseline to follow-up. Items for the questionnaires were taken from a previous diabetes study [39]. Baseline questionnaire (pre-test) included items on demographics, such as age, education, race, language of preference, country of birth, length of residency in the Valley, regular source of health care, and access to health care. Both questionnaires contained health behavior items, including two items on nutrition label literacy; an item assessing knowledge about interpreting serving sizes and knowledge of calorie intake based on serving size. Consumption of sugar-sweetened drinks was ascertained via four items, with two items, one for soda and another for non-soda, assessing how many ounces were consumed each day during the past 30 days and similarly two items assessing how often these drinks were consumed (e.g., a day, a week, a month). Two composite variables were then created to assess consumption of soda and non-soda sugary drinks per week. Fruit and vegetable (F&V) consumption was ascertained at baseline and follow-up using six items from the Thompson and Subar instrument [40]; three items for fruit consumption and three for vegetable consumption. Items asked how often the woman ate F&Vs (other than potatoes) during the past 30 days, the frequency (day, week, month) of consumption, and how many cups of F&Vs were consumed. Although the Thompson and Subar instrument used actual serving size, this was not easy for our participants to understand. Thus, we used ‘cups’ instead of serving size as this was familiar to participants. One composite variable for F&Vs was developed to measure the number of cups of F&Vs consumed per day for consistency across participants. Participants also were asked about food security; that is, how often they felt worried or stressed about having enough money to buy nutritious meals during the past 12 months. Finally, the frequency with which women ate food prepared at restaurants was assessed via two items; one item assessed how often, on average, the participant ate foods that were prepared in restaurants (no/yes; yes variable included ‘rarely’) and the second item assessed the number of times per week eaten food prepared in a restaurant among those who responded yes to prior question.

We captured amount of physical activity performed during leisure time using four items. Three items assessed the amount of physical activity performed by level of intensity (e.g., strenuous, moderate, or mild): ‘how many times per week do you perform physical activity for more than ten minutes during free time outside of work’. Another item assessed how often the participant engaged in regular physical activity long enough to sweat during the last seven days using a four-point Likert scale (never, rarely, sometimes, often).

Height and weight for participants at both time points used a SECA medical scale with stadiometer. CDC Standard weight status categories, developed by the Centers for Disease Control and Prevention, associated with BMI ranges for adults were used to calculate participant BMI categories (Normal: 18.5–24.9; Overweight: 25.0–29.9; Obese: 30.0+) [41]. We also assessed perceived self-weight at baseline and follow-up using one item asking about how the participant felt about her weight (overweight, slightly overweight, just about the right weight for me and slightly underweight).

### Data collection

Responses to all the questionnaires were collected and managed using REDCap electronic data capture tool. REDCap is a secure online software application designed to support and facilitate data capture for research studies [42]. The Fred Hutchinson Cancer Research Center Institutional Review Board approved this study (File #7293).

### Analytic plan

Quantitative data analyses were performed using STATA 14.2 (STATACorp, 2014). Study analysis focused on assessing workshop participation and comparing differences in obesity-related behaviors from baseline to follow-up. Descriptive statistics are presented using proportions of participants who completed the baseline and follow-up questionnaires. McNemar’s exact test for matched-paired binary data was performed to assess differences in marginal frequencies for nutrition label literacy, consumption of sugary drinks, and meeting national recommendations for F&V consumption. Paired-sample *t* test was performed to assess differences in mean consumption of F&Vs, performance of physical activity, and eating out at restaurants from baseline to follow-up. Differences in mean weight change and BMI from baseline and follow-up were also assessed.

## Results

### Participant characteristics

#### Demographics

A total of 50 women were recruited to participate in the pilot EHBA workshops, however, one woman self-identified as non-Hispanic White and was excluded from all analyses. All women included in this study (n = 49), self-identified as Hispanic; ages ranged from 19 to 75 years old (Table 1). Most participants (67.4%) reported having an education level ‘less than high school degree’. Among the women, 82.0% (n = 41) reported Spanish as their preferred language to speak and read and 82% completed the surveys in Spanish. One participant reported they preferred both English and Spanish. The majority of the participants were born in Mexico (70.0%) and reported living in the Valley for more than five years (79.6%). Over 40% (n = 21) had no health insurance of any kind.

**Table 1:** Participant demographics (N = 49)

	Total	%
Age		
18–34	15	30.6
35–44	19	38.8
45–64	14	28.6
65+	1	2.0
Education		
< HS degree	33	67.4
HS degree	8	16.3
> HS degree	8	16.3
Preferred language		
Spanish	41	82.0
English	9	18.0
Country of origin		
U.S	12	24.0
Mexico	35	70.0
Other	3	6.0
Length of time living in U.S		
< 1 yr	6	12.2
1 to 5 years	4	8.2
> 5 years	39	79.6
Health Insurance		
Private	10	20.4
Medicare	4	8.2
Medicaid	13	26.5
Other	1	2.0
No Health Insurance	21	42.9

### Health knowledge and behaviors

#### Nutrition health literacy

There was an increase in the number of women who demonstrated nutrition label literacy from baseline to follow-up. Specifically, there were 38 women at follow-up who interpreted calorie content per serving size in a container from the nutrition label correctly compared to 25 women at-time of baseline (77.6% vs 51.0%, respectively); further, the proportion of women who correctly identified daily calorie intake based on serving size increased from baseline to follow-up (76.1% vs 51.0%) (Table 2).

**Table 2:** Changes in the proportion of women with nutrition label literacy and food insecurity from baseline to follow-up

	Pre n (%)	Total # of respondents N	Post n (%)	Total # of respondents N	*p* value
Nutrition label literacy					
Correctly identified calorie content	25 (51.0)	49	38 (77.6)	49	.002
Correctly identified daily calorie intake	25 (51.0)	46	35 (76.1)	46	.043
Experienced food security	34 (69.4)	49	34 (69.4)	47	.569

#### Food insecurity

Of the women who responded to this question, 69.4% stated experiencing stress about having enough money to purchase nutritious foods at baseline and follow-up. Among the women who responded at both baseline and follow-up (n = 47), 74.5% had no change in their food insecurity status, 14.9% reported food insecurity at follow-up but not at baseline, and 10.6% reported food insecurity at baseline, but not after completing the EHBA workshop.

#### Consumption of sugar sweetened drinks

For soda and sugary drinks, 35.6% (n = 16) of the women reported a decrease in the number of ounces consumed per week from baseline to follow-up; however, 44.4% (n = 20) had no change and 20.0% (n = 9) reported an increase (Table 3). For non-soda sugary drinks, 37.8% (n = 17) of the women reported a decrease in the number of ounces consumed per week, 33.3% (n = 15) indicated having no change and 28.9% reported an increase. The average number of ounces consumed per week from baseline to follow-up decreased for both soda and non-soda sugary drinks (Table 4).

**Table 3:** Proportion of Hispanic women with changes in health behaviors and health status from baseline to post-EHBA Workshop implementation

	Decrease n (%)	Increase n (%)	No change n (%)
Nutrition			
Sugary drink consumption (soda)	16 (35.6%)	9 (20.0%)	20 (44.4%)
Sugary drink consumption (non-soda)	17 (37.8%)	29 (61.7%)	1 (2.13%)
F&V consumption	17 (36.2%)	17 (36.2%)	17 (36.2%)
Eating food prepared in restaurants	0	0	49 (100%)
Performed Physical Activity (PA)			
Leisure Strenuous	8 (17.0%)	13 (27.7%)	26 (55.3%)
Leisure Moderate	16 (34.0%)	17 (36.2%)	14 (29.8%)
Leisure Mild	15 (31.9%)	20 (42.6%)	12 (25.5%)
Engaged in regular PA enough to sweat	7 (14.9%)	26 (55.3%)	14 (29.8%)
Weight & BMI			
Weight (measured in pounds)	25 (54.3%)	18 (39.1%)	3 (6.5%)
BMI (kg/m^2^)	22 (54.3%)	19 (41.3%)	5 (10.9%)

	Better status	Worse status	No change
Perceived self-weight	8 (17.0%)	6 (12.8%)	33 (70.2%)

**Table 4:** Mean changes in health behaviors and health status from baseline to follow-up

	Pre			Post			*p* value
	n	Mean	(95% CI)	n	Mean	(95% CI)	
Nutrition							
Sugary Drinks (soda)^a^	49	1.53	(0.97, 2.10)	45	1.33	(0.82, 1.85)	0.517
Sugary Drinks (non-soda)^a^	49	1.96	(1.36, 2.56)	45	1.80	(1.21, 2.40)	0.682
F&V consumption^b^	48	3.67	(17.15, 34.22)	46	3.99	(20.33, 35.50)	0.293
Ate food from restaurants^c^	49	0.90	(0.81, 0.99)	48	0.90	(0.81, 0.99)	–
Physical activity							
Leisure Strenuous^d^	47	0.68	(0.27, 1.10)	47	1.10	(0.55, 1.66)	0.182
Leisure Moderate^d^	47	1.94	(1.24, 2.63)	47	2.02	(1.45, 2.60)	0.852
Leisure Mild^d^	47	2.26	(1.47, 3.04)	47	3.06	(2.26, 3.87)	0.112
Engage in regular PA enough to sweat^e^	47	1.13	(0.84, 1.42)	47	1.83	(1.52, 2.14)	< .001
Weight (measured in pounds)	49	168.8	(158.05, 179.49)	46	168.7	(157.90, 179.41)	.838
BMI (kg/m^2^)	48	30.2	(28.34, 31.98)	46	30.1	(28.30, 31.97)	.809

^a^Ounces consumed per week

^b^Number of cups of F&V consumed per day

^c^Number of women who ate food prepared in restaurants

^d^Times per week physical activity was performed

^e^Frequency of physical activity performed per week (never = 0, rarely = 1, sometimes = 2, often = 3)

– No difference in mean from baseline to follow-up

#### Fruit and Vegetable (F&V) consumption

Among our participants, 61.7% of the women increased the number of F&V cups consumed per day from baseline to follow-up, while 36.2% and 2.1% reported a decrease and no change, respectively, in the number of F&V cups consumed per day (Table 3). The mean number of cups of F&Vs consumed per day increased by 0.29 cups from baseline to follow-up (Table 4). A total of 53% of the women consumed the minimum F&V cups per day (i.e., 2.5) recommended for having a reduced risk of coronary heart.

#### Eating out at restaurants

From baseline to follow-up, the proportion of women who reported eating food prepared in restaurants did not change (Table 3). In addition, there was no difference in the mean number of women who reported eating food prepared in restaurants from baseline to follow-up (Table 4).

#### Physical activity

Changes in the number of times per week a woman reported performing physical activity from baseline to follow-up varied by intensity. At follow-up, 27.7% (n = 13) of women reported an increase in the number of times per week they performed strenuous leisure physical activity, 36.2% (n = 17) increased moderate leisure physical activity, and 42.6% (n = 20) increased their mild physical activity performed per week (Table 3). Among the participants, there were small increases in the mean frequency of physical activity performed per week from baseline to follow-up (vigorous: 0.68 to 1.10), moderate: 1.94 vs 2.02, and mild: 2.26 vs 3.06) (Table 4). There was also a significant increase from ‘rarely’ to ‘sometimes’ in the mean frequency women performed regular physical activity long enough to make them sweat in the last 7 days during free time (mean = 1.13, 95%CI: 0.84 – 1.42 to mean = 1.83), 95%CI: 1.52 – 2.14).

#### Weight and obesity status

We assessed weight at baseline (n = 49, mean = 167.5) and follow-up (n = 46, mean = 168.7), as well as changes in BMI for 46 of the women for whom we were able to capture this data at both time points. More than half of the women with complete data (54.3%) had a decrease in weight ranging from 0.2 to 11.4 lb, while 39.1% had an increase in weight (Table 3). There were no meaningful changes in mean weight and BMI from baseline to follow-up (Table 4). With regard to changes in perceived self-weight from baseline to follow-up, 70.2% reported no change, 17.0% reported a better self-perceived status, while 12.8% reported a worse status (Table 3).

## Discussion

This pilot study assessed the feasibility of implementing the *promotora*-led Eat Healthy, Be Active (EHBA) community workshops to improve nutrition label literacy and assist in the adoption of healthy behaviors among Hispanic women residing in a rural geographical area. The proportion of Hispanic women who demonstrated an ability to read nutrition labels accurately increased from 51% at baseline to more than 75% at follow-up, which is a positive predictor of dietary quality [43]. Among Hispanic populations, less than 20% demonstrate adequate comprehension of food labels [44]. Improving nutrition label literacy is an important step for addressing high obesity rates among Hispanic women since studies have found that individuals who check food labels when purchasing food are more likely to report having healthy dietary practices compared to non-label users [45]. For many Hispanic populations, low levels of numeracy and literacy may further limit their ability to comprehend complex nutrition labels [46]. Although, the EHBA workshops do not address health literacy or other factors associated with low educational attainment, the workshops provide an avenue for improving a Hispanic woman’s ability to understand useful information contained within food nutrition labels and to translate that into the management of obesity-related outcomes.

Sugar-sweetened beverages are major sources of added sugar in the diets of Americans, accounting for 36% of added sugar intake [47]. A higher consumption of sugar-sweetened beverages is associated with weight gain, risk of type 2 diabetes and hypertension [47, 48]. In our study, 16 women (35.6%) decreased their consumption of sugar-sweetened soda drinks over a six-week period, while 9 (20.0%) had an increase in the number of ounces of soda consumed per week from baseline to follow-up. However, among the women who had an increase, all women consumed less than 24 ounces of soda per week. Similar results were observed for non-soda sugary drinks. While the proportion of women who decreased their consumption of sugar-sweetened drinks may seem small, EHBA could assist Hispanic women in promoting a reduction in the consumption of such drinks overall. This is particularly important given that a reduction in consumption of one serving of sugary drinks per day has been shown to reduce blood pressure [48].

National organizations recommend making half of the plate F&Vs and choosing a variety of red, orange and dark-green vegetables for meals. Increasing the consumption of healthy foods is a main goal of the EHBA curriculum. Consumption of F&V cups per day increased among 29 women (61.7%). Although 17 women reported a decrease of F&V consumption per day, 35.3% of these consumed at least 2.5 cups of F&V per day. Evidence suggests that intake of at least 2.5 cups of F&Vs per day is associated with a reduced risk of coronary heart disease, including heart attacks and stroke [49]. Among the women in this study, the mean F&V consumption per day at baseline was approximately one cup higher than the F&V intake recommendations to reduce CHD.

The Physical Activity Guidelines for Americans recommends at least 75 min of vigorous intensity activity or 150 min of moderate intensity activity per week for adults [35]. The first five EHBA workshops include a one to two minute talking point about physical activity with weekly tips on how to incorporate physical activity into their daily routines. Workshop Six focuses entirely on educating individuals about the benefits of physical activity, provides recommendations for identifying and performing aerobic and strengthening activities, as well as assist participants in developing and sustaining a successful plan for performing daily physical activity. More than 25% of the women increased the number of times per week they performed leisure strenuous, moderate, and mild physical activity. After participating in the workshop, the mean number of times per week women performed strenuous and mild physical activity increased. There was a significant increase in the frequency a woman performed physical activity enough to make them sweat from rarely to sometimes. Therefore, EHBA could assist Hispanic women in identifying ways to increase their physical activity at home, even in areas with limited access to parks and community centers. Previous qualitative studies have identified lack of time, being tired and lack of self-discipline as barriers reported by Hispanic adults for not performing physical activity [50].

Similar to national obesity rates among Hispanics, 45% of the women participating in this pilot study were obese. Obesity was a secondary outcome of this study and, given the short study period of six weeks, we did not expect significant changes in weight or BMI from baseline to follow-up. However, there was a decrease in weight measured among 54.3% of the women, which also translated to a decrease in BMI. Among older women (e.g., 50 years of age and older), behavioral changes related to diet and exercise improves body weight and adiposity, especially if such lifestyle changes are maintained for over a year [51]. Other studies, such as the Diabetes Prevention Program (DPP) also provide strong evidence of the effectiveness of diet and exercise lifestyle interventions on decreasing incidence of obesity and chronic conditions over three years [51]. Thus, future implementation of EHBA curriculum should include additional follow-ups (e.g., 6-month and 12-month follow-ups) to assess sustained healthy behaviors and decreases in weight and BMI to better understand the long-term impact of EHBA participation on reducing BMI.

Inequities in access to healthy choices where Hispanics live and work contribute to the high rates of obesity among these populations [52]. Food insecurity was prevalent in this study, with 69.4% of the participants stating they felt worried or stressed about having enough money to buy nutritious meals. Future implementation of EHBA should be coupled with efforts that address social, environmental and financial barriers that limit access to healthy foods and places to perform physical activity. Strategies to address disparities in obesity rates among Hispanics must include sustained and community collaborative approaches that address the challenges that stem from social risk factors, which influence access to healthy and affordable food, as well as promote physical activity [52].

*Limitations*. This pilot study has several limitations. A total of 49 women participated in this study, which is a small number but consistent with the samples size for pilot studies [38]. There was variation in the number of women who responded to specific items in each questionnaire. Variation in the responses may be associated with factors unrelated to the actual content of the workshops, such as personal and cultural preferences to respond to specific items. In addition, information on nutrition and physical activity are based on self-reported data, which are susceptible to several types of biases (e.g., recall bias, measurement bias, and social desirability bias). For example, food frequency intake and amount of physical activity performed may be biased by a participant’s ability to recall actual behaviors in the last 30 days [53]. Participants may also respond to items in a manner that they may deem to be socially desirable, such as underreporting consumption of sugary sweetened drinks and/or overreporting intake of F&Vs and amount of physical activity performed [54]. Nonetheless, using self-reported questionnaire items to capture food intake and physical activity is a common method due to its reliability and validity in assessing behaviors (e.g., food consumption), low burden on participants, and ease of administering survey [54]. Although we did not have a control or comparison group, this is common in pilot studies. Future implementation of EHBA in rural Hispanic populations should include a larger sample size of women with comparison or control groups to assess the impact of EHBA. Additionally, the final assessment of the pilot study was at the end of the six weeks of workshops, which is a short time to achieve large improvements in health behaviors; it is important for a longer-term assessment of whether participants developed or maintained positive behavior changes over time.

## Conclusion

A *promotora*-led Eat Healthy Be Active community workshops can be feasibly implemented among Hispanic women residing in rural geographical areas to improve nutrition label literacy, as well as to promote behavioral changes that may be linked to obesity reduction. The average change in obesity-related behaviors from baseline to follow-up were in the appropriate direction. Larger studies with longer follow-up are needed to fully understand the impact of this curriculum on members of the Hispanic population in rural settings.

## Data Availability

The datasets used and/or analyzed during the current study may be available from the corresponding author on reasonable request.

## Abbreviations

NHW: Non-Hispanic white
EHBA: Eat Healthy Be Active
CHD: Coronary heart disease
CBPR: Community based participatory research
CAB: Community advisory board
CHE: Community health educator
F&V: Fruits and vegetables
